# Does mass drug administration for the integrated treatment of neglected tropical diseases really work? Assessing evidence for the control of schistosomiasis and soil-transmitted helminths in Uganda

**DOI:** 10.1186/1478-4505-9-3

**Published:** 2011-01-06

**Authors:** Melissa Parker, Tim Allen

**Affiliations:** 1 Centre for Research in International Medical Anthropology, Brunel University, UK; 2 Department of International Development, London School of Economics, UK

## Abstract

**Background:**

Less is known about mass drug administration [MDA] for neglected tropical diseases [NTDs] than is suggested by those so vigorously promoting expansion of the approach. This paper fills an important gap: it draws upon local level research to examine the roll out of treatment for two NTDs, schistosomiasis and soil-transmitted helminths, in Uganda.

**Methods:**

Ethnographic research was undertaken over a period of four years between 2005-2009 in north-west and south-east Uganda. In addition to participant observation, survey data recording self-reported take-up of drugs for schistosomiasis, soil-transmitted helminths and, where relevant, lymphatic filariasis and onchocerciasis was collected from a random sample of at least 10% of households at study locations. Data recording the take-up of drugs in Ministry of Health registers for NTDs were analysed in the light of these ethnographic and social survey data.

**Results:**

The comparative analysis of the take-up of drugs among adults revealed that although most long term residents have been offered treatment at least once since 2004, the actual take up of drugs for schistosomiasis and soil-transmitted helminths varies considerably from one district to another and often also within districts. The specific reasons why MDA succeeds in some locations and falters in others relates to local dynamics. Issues such as population movement across borders, changing food supply, relations between drug distributors and targeted groups, rumours and conspiracy theories about the 'real' purpose of treatment, subjective experiences of side effects from treatment, alternative understandings of affliction, responses to social control measures and historical experiences of public health control measures, can all make a huge difference. The paper highlights the need to adapt MDA to local circumstances. It also points to specific generalisable issues, notably with respect to health education, drug distribution and more effective use of existing public health legislation.

**Conclusion:**

While it has been an achievement to have offered free drugs to so many adults, current standard practices of monitoring, evaluation and delivery of MDA for NTDs are inconsistent and inadequate. Efforts to integrate programmes have exacerbated the difficulties. Improved assessment of what is really happening on the ground will be an essential step in achieving long-term overall reduction of the NTD burden for impoverished communities.

## Introduction

Global aspirations to achieve the Millennium Development Goals by 'making poverty history' and alleviating the suffering of 'the bottom billion' has generated unprecedented attention on a range of predominantly parasitic and bacterial infections endemic in large parts of sub-Saharan Africa. These infections cause significant morbidity, if not mortality, among politically and economically marginal populations They are increasingly grouped together and referred to as 'neglected tropical diseases' [NTDs]; and substantial funding and a range of medicines have become available from international agencies and pharmaceutical companies to support the design and implementation of national programmes seeking to control them. All programmes involve the mass distribution of drugs, free of charge, to adults and children living in areas where the diseases are endemic.

A great deal of optimism surrounds mass drug administration [MDA] programmes. Numerous publications emphasise their potential to alleviate sickness and suffering (see, for example, Brady et al [1]; Engel et al [2]; Fenwick et al [3]; Fenwick [4]; Molyneux et al [5]; Molyneux et al [6]; Manderson et al [7]; Hotez [8]; Hotez et al [9]) and the majority of reported results are presented in a positive light (see, for example, Kabatereine et al [10]; Kabatereine et al [11]; Knopp et al [12]; Standley et al [13]; Yu et al [14].) In its first major report on NTDs, published in 2010, the Director-General of the World Health Organisation claims that nearly 670 million people had been reached with preventive chemotherapy by the end of 2008. She maintains that aiming at complete control of NTDs is fully justified, and that there is a 'possibility that several of these ancient diseases could be eliminated by 2020' if current efforts to scale up interventions are increased [[15]:v].

There are, however, a number of academics who have expressed concern about the way in which MDA for NTDs is being implemented. For example, Katabarawa et al [16]; Katabarwa and Richard [17]; Amazigo et al [18] and Parker et al [19] have documented the hazards of relying upon unpaid volunteers to distribute drugs within designated communities, while Gryseels et al [20], and Coulibaly et al [21] suggest that vertical systems of drug administration run the risk of overwhelming fragile health-care delivery mechanisms. In addition, Utzinger et al [22] questions the sustainability of control programmes that rely so heavily upon preventive chemotherapy; and Kolaczinski et al [23], aware of the rhetoric surrounding the benefits of MDA control programmes, calls for a more adequate evidence base. The article also draws attention to the point that current endeavours to 'integrate' vertical control programmes may lead to the development of a parallel health-delivery system, with potentially serious consequences for ministries of health. A rather different issue has been highlighted by the work of Parker et al [19]: namely, that it is hazardous to assume that targeted populations will necessarily understand or agree with the rationale for MDA. Indeed, they may actively resist treatment. This can have negative ramifications, not only for the control of NTDs, but also for other infectious disease control programmes. Finally, Danso-Appiah et al [24], Lammie et al [25], Fenwick [4] and Gryseels [26] have highlighted limits in knowledge surrounding the safety and efficacy of combining drugs for some NTDs, with Gryseels taking the argument a step further and raising concerns about possible drug resistance if mass treatment becomes long term.

The literature highlighting reservations about NTD programmes is not cohesive. Indeed, some of the authors cited above fundamentally disagree with each others' interpretations of the available evidence - most notably on the issue of drug resistance (see, for example, Gryseels [26] and Tchuente et al [27]. Also, in spite of their doubts, most remain very committed to the idea of MDA for NTD control. Nevertheless, taken together, the insights and arguments they present raise important questions about current approaches. Although the point is not always made explicit, there is an underlying recognition that some assessments have been shaped by the need to raise and sustain large amounts of funding, and have been overly positive. Such exaggerations may prove counter-productive.

A danger now is that there will be a backlash, and that genuine achievements will be overlooked: a point that has been made recently by Narcis Kabatereine, Uganda's head of vector control. He has dedicated his working life to the control of schistosomiasis, is a co-author of several of the papers cited above and below extolling the virtues of MDA, and has been hailed as the 'unsung hero of neglected tropical diseases' for his dedication and commitment to the cause [28]. However, he has become worried about the way in which external funding from the United States Agency for International Development [USAID], channelled through the American organisation RTI International, is hindering the development of a fully-integrated national control programme for NTDs. Instead, USAID are developing a separately funded, parallel structure located within the Ministry of Health. In a recent interview he has gone so far as to not only draw attention to our own findings that suggest that recent efforts to control schistosomiasis have faced problems [19], but also to express the view that current approaches prohibit addressing difficulties with the roll out of MDA and that this could well have a negative impact on drug uptake in the future [28]. His concerns are reflected in a carefully worded paper which he has co-authored with colleagues involved in the establishment of integrated control programmes in Southern Sudan, Mozambique and Tanzania [29].

The findings presented in this paper explore these issues. Drawing on local level research undertaken in Uganda since 2005, it shows that NTDs can potentially be controlled by MDA, but not all that has been claimed about successful treatment coverage is based on robust evidence. The problems arising on the ground are often acute. The paper goes on to suggest that a change in direction is required if targets are to be met and reductions in NTD prevalence maintained. Among other things, it demonstrates the need for more appropriate methods of drug delivery, improved communication with target populations, and a more rigorous approach to monitoring. Insights derived from fieldwork at a variety of locations spread across Uganda demonstrate how lessons could be learned. Comparative results from similar research carried out in Tanzania are briefly reviewed in the conclusion.

Uganda is an interesting and important place to explore social responses to MDA, not least because it was the first country to establish national control programmes for the control of NTDs. More specifically, national control programmes were established for onchocerciasis, lymphatic filariasis, schistosomiasis and soil-transmitted helminths in the early part of 2000. The desire to integrate programmes and thereby avoid the unnecessary duplication of resources led to the establishment of the National Control Programme for the Integrated Control of Neglected Tropical Diseases in Uganda in 2007. Since then, in areas of co-endemicity, endeavours have been made to deliver free treatment for onchocerciasis, schistosomiasis, lymphatic filariasis and soil-transmitted helminths simultaneously.

## Recent assessments of MDA for schistosomiasis and soil-transmitted helminths in Uganda

As elsewhere, published epidemiological and parasitological research on Uganda suggests that mass treatment has successfully reduced the prevalence and intensity of infection, as well as clinical indicators of morbidity among school children in targeted areas [10,11]. However, there are limitations with this type of work. Kabatereine et al's research [11], for example, attempted to monitor cohorts of children that had received annual rounds of praziquantel (for the treatment of schistosomiasis) and albendazole (for the treatment of soil-transmitted helminths). Follow up visits occurred six months after each round of treatment and every time a positive case was detected, additional treatment was provided. As a result, the cohort studies provide a picture of what happens when treatment is made available at six monthly intervals. They do not capture the experiences of children that are treated at 12 monthly intervals by control programmes, nor do they shed light on the proportion of school-aged children that receive multiple rounds of treatment. This is unfortunate as the number of children successfully completing primary school in under-resourced, rural areas is often small, a point underlined by the large number of children that have dropped out of cohort studies. Thus, cohort studies are biased towards children from families of long-term and stable residency who are known to receive multiple rounds of treatment.

Research undertaken by Brooker et al [30] also leaves some important questions unanswered. This cross-sectional study used the Lot Quality Assurance Sampling (LQAS) technique to map the prevalence of Schistosoma mansoni among school children in four different regions in Uganda, before and after several rounds of mass treatment in 2003 and 2006 respectively. Working with four teams of three technicians, they sampled an average of 8 schools a day for 3 days in Eastern, Central, Northern and Western regions of Uganda. Fifteen school children were randomly selected from each school; and the prevalence of S.mansoni was deemed to be low if 2 or less children tested positive for S.mansoni and high if 7 or more children tested positive for S.mansoni. GPS readings for each school were also recorded and this allowed the creation of prevalence maps using GIS software. The results are visually impressive and have fed into the general optimism about the 'magic bullet' nature of NTD control. However, extrapolating from such a small sample is problematic, especially as other data collected at schools in some of the sampled districts suggests that levels of re-infection are high. For example, rates of re-infection among school children were recently reported to be 54%, 84% and 60% at Panyimur Primary School, Nyakagei Primary School and Boro Primary School in Panyimur sub-county, Nebbi district (Schistosomiasis Control Initiative [SCI] Country Programme Manger, personal communication, 2008). In short, the suggestion on the SCI website (http://www.imperial.ac.uk/schisto, accessed on November 15^th ^2008) that the rapid mapping exercise illustrates that there is a "realistic chance of bringing bilharzia infection in Uganda to below an intensity level at which it is a major public health problem" is surely open to question.

In addition to epidemiological and geographical surveillance data exploring the impact of MDA for schoolchildren in Uganda, endeavours have been made by Lubanga [31] and Fleming et al [32] to monitor social responses to MDA among adults in Uganda. The type of research undertaken by Lubanga is often referred to as 'qualitative research' or a 'knowledge, attitudes and practice (KAP) survey', while Fleming et al [32] refer to their work as 'process monitoring'. Useful insights may emerge from this type of work, but a persistent problem with both approaches is that findings from semi-structured questionnaires, focus group meetings and key informant interviews are stripped of their political and social context and presented as a series of disembodied quotes. More often than not, they are taken as unproblematic statements that reveal the 'true' state of play, and no attempt is made to corroborate ideas with information derived from other methods or data sources.

Lubanga's research, for example, explores social responses to MDA in Nebbi and Busia districts, Uganda. It presents a series of quotes derived from focus groups and interviews to suggest generally successful drug take-up and even a growing demand for treatment. No attempt is made to explore the generalisability of findings by undertaking surveys of drug uptake among targeted populations in the selected districts; or to analyse the data in relation to quantitative information monitoring the take-up of drugs in Ministry of Health registers; or to observe what actually happens when drugs are distributed at a local level. As a result, it is not possible to know if those stating that they had received drugs had actually swallowed them, or if perspectives differed significantly between research sites. Nevertheless, the information presented in Lubanga's report was subsequently used as evidence in the article published by Fleming et al [32]. Here, findings from Lubanga's KAP survey are uncritically combined with data documenting the total number of drugs sent out from Kampala. Again, the paper does not discuss how the drugs were actually distributed between and within districts; or whether or not they were swallowed, in whole or part, by targeted people once they had been distributed at a village level. It may be that the positive assessment of MDA presented in this paper is correct, but it is not really possible to come to such a conclusion from the findings presented.

However, limitations in the work cited above are relatively minor compared to USAID funded assessments by RTI International. Monitoring undertaken since 2008 suggests, for example, that USAID/RTI endeavours to take primary responsibility for the roll out of mass treatment for NTDs has been extremely successful. That is, the NTD Control Programme had provided, by the third year of the programme, 27,656,133 treatments to 13,687,841 people in 72 districts within Uganda (http://ntd.rti.org, accessed on 28^th ^September 2010.) A map is also shown on the RTI website to convey a sense of a hugely successful programme; and links are provided to reports describing the achievements of community drug distributors and treatment programmes in formerly war-affected parts of the country. Precisely how these facts were collected is not made clear, but from our own observations and discussions with staff in the field, they appear to be derived from short questionnaires carried out on a small number of people at selected districts, combined with records relating to the overall numbers of tablets sent out from the national headquarters. There is no indication to suggest that any effort was made to look beneath the surface and to see how stated practice related to actual behaviour. The absence of critical discussion of findings and the way in which they have been presented suggests that the primary purpose is to 'prove' the on-going success of the programme with a view to justifying the next round of USAID funding.

In short, biomedically-orientated and programme-linked assessments have, to date, focused on children rather than adults and while some of the findings emerging from this type of work in Uganda are interesting, they leave the following questions unanswered: What proportion of adults living in endemic areas are receiving drugs from MDA programmes on an annual basis? How are these free drugs perceived? Are they being swallowed? Do local understandings of the aetiology and treatment of 'NTDs' influence the consumption of drugs? Are there any indications to suggest that adults are becoming aware of the benefits of treatment? These are important questions to address as the success of the National Control Programme ultimately depends upon adults appreciating the benefits of treatment to a point where they become willing to purchase the drugs for themselves and their children when free treatment comes to an end.^1 ^As indicated above, a small amount of research exploring social responses to MDA among adults has purported to answer some of these questions, but the historical, political, economic and social context of treatment has been ignored, and findings are neither assessed critically nor adequately triangulated.

The research presented here attempts to reverse this trend. It builds upon the article by Parker et al [19], which raised serious concerns about MDA based on findings from two districts. Key points are updated in this paper and compared with results from other parts of Uganda. It is demonstrated that, while the programme for the Integrated Control of Neglected Tropical Diseases has a clear protocol for rolling out drugs in similar ways across the country, in practice distribution varies considerably from place to place. A range of factors at a local, national and international level affect the uptake of drugs between and within districts, and even within villages. These findings have wide-ranging implications, not only for those committed to the control of NTDs in Uganda and other parts of East Africa, but also for furthering our understanding of the role public policy can play in alleviating sickness and poverty more generally.

## Fieldsites

Research took place in three districts in north-western Uganda and two districts in south-eastern Uganda. In north-western Uganda, the selected districts were Nebbi, Moyo and Adjumani (which formerly comprised part of Moyo district). Nebbi district shares a border with the Democratic Republic of Congo, while Moyo and Adjumani districts share their northern borders with Sudan. In south-eastern Uganda, research was undertaken in Busia and Bugiri districts. Busia district shares a border with Kenya, while Bugiri district borders Busia. Both districts have villages located on the shores of Lake Victoria. The populations inhabiting these five districts have all been influenced, in different ways, by the changing political and economic landscape of Uganda. Indeed, MDA is occurring in places that could not be more different from each other and, as will be explained below, that is one reason why take up has varied so dramatically.

## Methods

Fieldwork took place over a period of four years between 2005 and 2009. At all field sites, research focussed on the roll out of MDA for schistosomiasis and soil-transmitted helminths and, where relevant, lymphatic filariasis and onchocerciasis.^2 ^While the research methods at these sites involved looking at all kinds of available evidence, particular weight was given to the following: (1) assessments of the local context in which MDA is occurring, including historical factors and the current political and economic situation; (2) data recording the names and number of adults in Ministry of Health registers who were reported to have received drugs for schistosomiasis, soil-transmitted helminths and, if appropriate, lymphatic filariasis and onchocerciasis; (3) observations of MDA as it occurred; and (4) survey data (involving a random sample of at least 10% of households at each location) recording the proportion of adults who reported having been given the opportunity to receive and swallow drugs for the treatment of schistosomiasis and soil-transmitted helminths and, where relevant, lymphatic filariasis and onchocerciasis. In addition, (5) open-ended and semi-structured interviews were undertaken with those involved in or affected by MDA, including political figureheads, religious leaders, local healers (spirit mediums, herbalists), fisherman, market women and farmers. In short, every effort was made to triangulate information and to use a variety of methods to cross-check results.^3^

Inevitably, there were some variations in the methods employed between sites, notably with respect to the use of registers. In some placers, these were found to have been lost or were so poorly kept as to be almost useless. In other places, notably Nebbi district from 2004-2005, the data recorded was detailed and proved to be accurate when cross-checked with survey information on drug take-up. Variations in data used between sites are detailed in the sub-sections below.

### Methods in Panyimur sub-county, Nebbi District

A total of 7 months fieldwork was undertaken In Panyimur, Nebbi district between May 2005 and September 2008. This was the first research site, and the most intensively studied. In 2005, participant observation, open-ended, unstructured interviews and semi-structured interviews were undertaken over a period of four months, and full details of this research have been published by Parker et al [19]. Further research was undertaken from July to September 2008, with the assistance of four MSc students from the London School of Economics and five locally-trained researchers, three of whom assisted with the research in 2005.

In common with the research undertaken in 2005, a variety of methods were drawn upon to explore local responses to mass treatment in 2008. These include the following: participant observation in Panyimur Trading Centre; 50 open-ended, unstructured interviews and 10 focus group discussions with district officials, local council officers, teachers, healers, politicians, market traders and groups of migrants. Semi-structured interviews were also undertaken in 15 of the 49 villages that constitute Panyimur sub-county. These interviews occurred with a 10% random sample of adults that were registered by staff from the Ministry of Health in MDA treatment registers in 2004; and they were selected to assess, among other things, the proportion of people that had been re-treated in 2007, compared to 2004 and 2005.

In addition, research was undertaken to explore the proportion of adults receiving drugs for NTDs who were migrants, rather than residents. To this end, a five percent random sample of the names of adults who took drugs during the mass distribution in 2007 was taken from the Ministry of Health registers documenting the uptake of drugs in five selected villages: Singla Central, Dei B, Afhoda, Awulu, and Lwalla.^4 ^This five percent sample consisted of 50 names; and the names were then cross-checked with the household register kept by the local council chairman (LC1) for the village. To further establish the mobility status of each of the drug recipients on the list, an endeavour was made to not only contact the local council chairman but also friends, neighbours and relatives within the village to try and find out the various occupations and travel patterns of each person on the list.

### Methods in Moyo and Adjumani Districts

Fieldwork on NTDs was undertaken for the first time in Moyo and Adjumani districts in 2008. That is, 103 semi-structured interviews were undertaken in 7 villages on or close to the River Nile in Adjumani district (Adjujo, Agiforo, Arra Central, Maaji, Malhewe internal displacement camp, Ogolo internal displacement camp and Pamajua) and 72 semi-structured interviews were undertaken in 5 villages and 2 trading centres in Moyo district (Olia West, Pakaa West, Pakonira East, Panjala, Paubu, Dufile, Laropi). These semi-structured interviews were supplemented with open-ended interviews covering a range of issues with village drug distributors, district vector control officers and supporting staff, local healers and political figureheads. Moreover, research in Moyo and Adjumani districts benefitted from the fact that one of the authors, Tim Allen, had undertaken long term ethnographic fieldwork on health and healing in the region in the 1980 s and 1990 s [33-38]. This research involved the acquisition of detailed information about many aspects of social, political and economic life and proved enormously helpful when interpreting information arising from semi-structured interviews.

### Methods in Busia District

Research was undertaken in Lumino sub-county for the first time between July-August 2009. In addition to participant observation fieldwork at landing sites on the shores of Lake Victoria, open-ended, unstructured interviews were undertaken with staff representing the Ministry of Health in Busia District as well as drug distributors, labourers, fisherfolk, political figureheads, clan leaders, school teachers, religious leaders etc. Semi-structured interviews were also undertaken with a 10% random sample of adults residing in 14 of the 64 villages that constitute Lumino sub-county. The selected villages were from five of the six parishes in the sub-county, and they included a range of villages located by rivers and Lake Victoria, as well as villages with trading centres and villages relying almost exclusively on agricultural production.

## Results

### Self-reported uptake of drugs among adults

The data presented in this section focuses on self-reported uptake of drugs among adults in Nebbi, Moyo, Adjumani and Busia districts. Wherever possible, findings are cross-checked with data recording drug uptake in Ministry of Health registers. Praziquantel has been distributed regularly in all four districts for the treatment of schistosomiasis since at least 2004, and albendazole has been the frontline treatment for soil-transmitted helminths. By contrast, ivermectin has been distributed on an annual basis in Moyo and Adjumani districts since the early part of 2000 and it has also been distributed in Panyimur, Nebbi district since August 2008. There were no plans to distribute the drug in Busia district as lymphatic filariasis and onchocerciasis are not endemic in this part of the country. Results are presented on a district by district basis and demonstrate that self-reported uptake of drugs among adults varies considerably between and within districts.

#### Self-reported uptake of drugs in Panyimur sub-county, Nebbi District

To start with Panyimur: it was possible to use Ministry of Health registers in Panyimur to gauge the proportion of adults that were re-treated for schistosomiasis and soil-transmitted helminths in 2007, compared to 2004 and 2005. This was because the 2004 Ministry of Health registers provided a detailed record of the names of all adults and children in each village, irrespective of whether or not they had received MDA. Semi-structured interviews were then undertaken with a 10% randomly selected sample of the adult population, from these registers, in 15 of the 49 villages that constitute Panyimur sub-county. To ensure that a broad range of villages in the sub-county were selected, five villages were selected from each of the three parishes that make up Panyimur sub-county. The selected villages in Boro Parish were: Kaligonzi, Nyakiro, Ayago, Situ and Wankado East. In Nyakagei Parish, the villages selected were: Dei central, Adondo, Kimor, Lwala and Opurukojo; and the selected villages in Ganda Parish were Kulluber, Munyua, Mukindwa, Singla B and Kidi Achoka.

Table 1 shows that the number of adults receiving treatment in 2007 (compared to 2004) declined in all three parishes, with the decline being particularly marked in Nyakagei and Boro Parishes. In all three parishes, drug uptake fell short of the target of reaching 75% of the adult population.

**Table 1:** Self-reported uptake of drugs for schistosomiasis and soil-transmitted helminths among adults in selected villages, Panyimur, Uganda

	2004	2005	2007
Ganda Parish N = 148	62%	36%	59%

Nyakagei Parish N = 192	65%	43%	36%

Boro Parish N = 255	78%	32%	33%

#### Self-reported uptake of drugs in Moyo and Adjumani Districts

In common with Panyimur sub-county, a semi-structured interview was undertaken with a 10% randomly selected sample of the adult population in seven villages in Adjumani district, and five villages and two trading centres in Moyo district. All selected villages and trading centres were located within 2 kilometres of the River Nile.

With respect to Moyo district, self-reported drug uptake among adults was variable. Table 2 shows, for example, that the uptake of drugs in three of the seven villages was 90% or more in 2008 whilst the other four villages had an uptake of 50% or less (and the overall average in Moyo was 55%). The years 2007, 2006 and 2005 also witnessed considerable variation. That is, the overall uptake of drugs in 2007, 2006 and 2005 was 41%, 39% and 32% respectively, with uptake ranging from 0-95% in 2007, 8-76% in 2006 and 8-50% in 2005. In 2007, two out of the seven selected villages reached or exceeded the target of treating 75% or more of the adult population.

**Table 2:** Self-reported drug uptake as a percentage at selected villages in Moyo District, 2005-2008

	Olia West	Pakaa West	Pakonira East	Panjala	Paubu	Dufile	Laropi
2008	90	50	92	5	100	15	33

2007	0*	17	38	95	17	88	33

2006	50	8	38	32	17	54	76

2005	30	8	38	26	50	46	29

* No reported distribution

Results from Adjumani district are more promising, with overall uptake increasing from 29% in 2005 to 59% in 2006 and 50% in 2007. In 2008, the reported uptake increased further to 74%. In common with Moyo district, there was considerable variation between selected villages. That is, the uptake of MDA in 2008 ranged from 5% to 100%, with 5 of the 7 villages exceeding the target of 75% uptake among adults. These data are presented in Table 3. Unfortunately, it was not possible to use information in the Ministry of Health drug registers to corroborate or refute these data as the information had not been recorded in a usable fashion (a point discussed below).

**Table 3:** Self-reported drug uptake as a percentage at selected villages in Adjumani District, 2005-2008

	Adjujo	Agiforo	Arra Central	Maaji	Malehwe IDP*	Ogolo IDP	Pamajua
2008	89	94	5	81	100	70	79

2007	33	50	53	19	78	86	37

2006	78	39	68	78	33	47	74

2005	22	6	42	37	11	32	58

* Internal Displacement Camp

#### Self-reported uptake of drugs in Busia District

The analysis of survey data documenting self-reported uptake of drugs among adults for the treatment of schistosomiasis and soil-transmitted helminths suggests that 64% of those interviewed received praziquantel in 2009 and 67% received the drugs in 2008. The proportion of adults swallowing the drug under the direct gaze of the drug distributor was 50% in 2009 and 58% in 2008. While it proved impossible to acquire detailed information on drug uptake prior to 2008, information emerging from semi-structured interviews and key informants indicated that uptake had fallen in 2008 and 2009 due to increasing food shortages within the district, and a reluctance to swallow praziquantel if there was insufficient food to mitigate the side-effects. Nevertheless, these data suggest that while the majority of adults have not received three or more rounds of praziquantel since 2004, a mere 18% have never received any treatment. Before discussing explanations for variations in the uptake of drugs between study sites, it is important to first reflect on the quality of data assessing adult responses to MDA.

#### Interpreting data on the uptake of drugs among adults

At all field sites, it proved extremely difficult to acquire a detailed understanding of the proportion of the adult population that had taken praziquantel and/or albendazole and ivermectin in any one year. With the exception of Panyimur sub-county in 2004 and 2005, the Ministry of Health registers recorded, on a village by village basis, the names of all the people that were given the tablets as well as their ages, height and the number and types of tablets they were given. Names of all residents in villages receiving MDA were not entered prior to treatment nor were they entered on a household by household basis. In the absence of reliable census data, it is thus impossible to use 'official' data to gauge the proportion of the adult population treated or the proportion of adults re-treated.

A second difficulty concerns the interpretation of data in the Ministry of Health registers as the mode of distribution has changed over time. Even in Panyimur sub-county, where there is a possibility of comparing the proportion of adults that have received treatment at different points in time, it is not straightforward. That is, drugs were distributed from door to door in 2004 and 2007, and adults were able to take them in their own time. In 2005, however, villagers had to go to a central distribution point and swallow the drugs under the direct gaze of the drug distributors if they wanted to receive praziquantel and/or albendazole. While a few exceptions were made, it meant that those recorded in the drug distribution register as taking the drugs had actually swallowed them. The same cannot be said for those recorded in the Ministry of Health registers for 2004 or 2007. In other words, the decline in the uptake of drugs in 2005 may not be as marked as the official figures suggest as the uptake of drugs in 2004 and 2007 may be artificially high.

A third difficulty concerns the proliferation of Ministry of Health registers. From 2004 to 2007, there were two sets of registers in circulation: one documented the uptake of drugs for the treatment of schistosomiasis and soil-transmitted helminths, while the other documented the uptake of drugs for lymphatic filariasis and onchocerciasis. In 2008, new 'integrated treatment' registers arrived with a view to monitoring the uptake of drugs for schistosomiasis, soil-transmitted helminths, lymphatic filariasis, onchocerciasis and trachoma. This register was meant to replace existing registers from July 2007, but it arrived late. This, in itself, created confusion - with some drug-distributors over-writing the 2007 and 2008 columns with 2008 and 2009 respectively, while others did not.

In Moyo and Adjumani districts, the situation was complicated by the fact that the registers documenting the uptake of drugs for ivermectin and albendazole were not withdrawn. This generated enormous confusion as some drug distributors decided to record the names of those receiving ivermectin in the old Carter Centre/Ministry of Health registers; while others wrote the same name in two registers (i.e. the Carter Centre/Ministry of Health register and the new USAID/RTI/Ministry of Health register); and still others felt so overwhelmed by the volume of work that they simply handed out drugs without recording any information in either register. Integration of treatment in this respect has failed as it has led to the collapse of an already fragile mechanism for MDA accounting. From a research and monitoring point of view, it renders the official registers almost useless.

A fourth problem is that the majority of drug distributors handed out drugs without observing their consumption. This compounds a fifth problem, which concerns the interpretation of data documenting the uptake of drugs from semi-structured and open-ended interviews with MDA recipients. There are several aspects to this, all of which tend to over-estimate rates of self-reported drug consumption. It was common at most sites for those interviewed for the first time to say positive things about treatment and to ask for more tablets. Yet, some of these same people would say something rather different when interviewed again some days or weeks later. In Panyimur in 2005, several respondents who had been very positive about MDA were observed not accepting the drugs during distributions, or taking them, but refusing to swallow them. While there is no doubt that drugs are being distributed at a village level and in several studied locations, the majority of adults are being given the opportunity to take them, it does not follow that they are being consumed, or consumed in the right doses. In Panyimur, where the language spoken is Alur, words for 'to take' and 'to swallow' are 'nitingo' and 'mwonyo' respectively, but if you ask the question 'did you take the tablets?' and an informant answers 'yes, I took the tablet', that could mean 'yes, I took the tablet and swallowed it' or 'yes, I took the tablet, and put them in my pocket.'

Great efforts were made to clarify what actually happened, but it was hard to know at what point a really straightforward answer was being given. Even informants who came to know the researchers really well appeared reluctant to say that they had not swallowed the tablets for fear of either being asked to return them or being forced to swallow them under the gaze of the interviewer. Neither would, of course, have happened, but the fear was palpable, particularly if someone had experienced side effects in the past. There were numerous occasions when informants eventually admitted that they had been given the tablets but kept them or taken the tablets and only swallowed some of the them. The following comment by a 43 year old woman, living close to Lake Albert, illustrates this point. She said: "I took two tablets [but] I have kept the rest for when I fall sick."

In Moyo and Adjumani districts, a further difficulty with interpreting data on the uptake of drugs, is that increasing numbers of people are not sure what drugs they are swallowing. Ivermectin is a small, white tablet; praziquantel is long, white and has two horizontal marks on it; and albendazole is white, with one horizontal mark on it. It is not quite as long and does not smell in the way praziquantel smells, but the differences are subtle and easy to confuse. While every effort was made to help informants differentiate between these two tablets, there were numerous occasions when adults shook their heads, unable to speak with confidence as to whether they had taken ivermectin and albendazole or ivermectin and praziquantel or ivermectin, praziquantel and albendazole. Where this occurred, the method adopted in this research was to call over neighbours and to have a collective discussion to help clarify the issue. Such discussions were usually interesting in themselves and, in each case, a consensus view was taken on which drugs had been distributed on which occasion and whether or not they had been taken by the individual concerned.

Some adults also struggled to recall the precise year(s) in which they had received and/or swallowed drugs - though this was rarely an issue in the northern districts of Nebbi, Moyo and Adjumani as the distribution of tablets was 'news' at all field sites, and the vast majority of people had clearly discussed whether or not to receive and/or swallow the relevant tablets with relatives and friends. In other words, it was not something one could forget too easily. However, it was more of a problem in Busia district, south-eastern Uganda where MDA is one of many programmes run by the government and/or NGOs. Again, where there was confusion about years of distribution, this was resolved in discussion with neighbours and reference to the relevant drug distribution registers.

In spite of the difficulties in eliciting accurate self reported information about drug take up, and the need to triangulate findings with other data wherever they were available, it is apparent from the findings presented above that the integrated national programme for NTD control has heterogeneous effects. To be more specific, the uptake of drugs among adults is low in Panyimur, variable in Moyo and Adjumani districts and relatively high in Busia district. Why? These questions are addressed, in turn, in the following section.

## Discussion

### Why is the uptake of drugs among adults low in Panyimur?

A range of political, economic and social factors influence the uptake of drugs in Panyimur. These include a fear of treatment, divergence between local and biomedical understandings of NTDs, insufficient and inadequate health education, migration patterns, ineffective systems of drug distribution and the changing political and economic context in which MDA occurs. These issues are discussed in turn.

#### Fear of treatment

In common with 2005, the majority of informants (70%) throughout the sub-county expressed a fear of treatment. This fear took a variety of forms, including a fear of side-effects arising from treatment with the drug, praziquantel; a fear of infertility and/or a fear of death. A 35 year old male fisherman, for example, said:

"I found my child under a tree ... her clothes covered in diarrhoea and vomit. Then I hear she has taken those drugs at school ... aarh! I do not want my children to take them next time. I shall not take them anymore."

Similarly, a 27 year old male from Sendi village, Panyimur sub-county captured some of these anxieties when he said: "In Dei, a woman was tall and thin and given too many drugs. She died so some people fear the drugs." This comment was followed by a discussion of mass treatment in which he mentioned that he had been given the drugs, praziquantel and albendazole, three times in 2005, 2006 and 2007. The first time, he took four tablets in one go and fell ill for two weeks. As a result he lost his job and he now feels afraid to take them again. The four tablets he was given in 2007 are still in his house and he expressed uncertainty as to whether he should take them. However, he also reported having pains in his stomach so he was turning over in his mind the possibility of taking them once he had planted the cotton, but emphasised that if he took them then he would only take one tablet at a time, rather than all four together.

Without a doubt, the occurrence of side-effects generates confusion and anxiety, and a fear of treatment contributes to the low uptake of drugs in Panyimur sub-county. In contrast to 2005, however, informants were less likely to use these fears to question the intentions of government and donors. Instead, their questions suggested that at least some adults are becoming accustomed to 'strong' drugs. The following quotes illustrate this point:

"Why are the drugs [praziquantel] weak these days? At the start [2004], it was strong and had bad side-effects .. does it mean it is not working anymore?" (53 year old male from Ganda Parish.)

"This time we did not get any side-effects. It means the drugs are useless and not working anymore ... other types of drugs should be brought" (30 year old female from Singla B, Ganda Parish)

#### Divergence between local and biomedical understandings of NTDs

In north-western Uganda, local understandings of '*bilharzia*' do not mirror biomedical understandings of 'bilharzia' and intersects with other local afflictions. In Panyimur, a local affliction known as '*awola*' mirrors the signs and symptoms of those associated with infection by S.mansoni. In 2005, the diagnosis of *awola *was often cited as a reason for rejecting treatment as it was widely believed that if a person with *awola *swallowed praziquantel, they would die. By 2008, *awola *was no longer so closely associated with the signs and symptoms of *bilharzia *and it did not, therefore, impinge on treatment in the way that it had done previously. Indeed, many local healers who were interviewed in 2008 said they had taken the tablets themselves. Also, the local affliction, *awola*, became associated with other diseases. It is impossible to quantify these changes, but it is striking that none of those interviewed in 2008 stated that a person with *awola *would die if they swallowed praziquantel, whereas this was almost universally asserted in 2005.

#### Insufficient and inadequate health education

In common with 2005, health education remains inadequate. To our knowledge, endeavours to provide information about the aetiology of NTDs and the rationale for mass treatment is restricted to district staff running training days with school teachers and drug distributors. At best, these sessions involve discussing how to administer the drugs safely, but key questions are not adequately addressed. For instance, why do some people suffer serious side-effects and other people not suffer any? Why should people who feel well take the drugs? Why is it necessary to allocate drugs by height rather than weight? What is the point of taking the drugs, when re-infection is likely? What diseases do the drugs treat? How do biomedical understandings of the aetiology and treatment for NTDs relate to local understandings and therapies for these diseases? It would be really helpful to address these questions, especially as the majority of adults do not perceive bilharzia (S.mansoni) and soil-transmitted helminths to be major health problems. Malaria, HIV/AIDS, poisoning and witchcraft are thought to be much more serious. In fact, the absence of information about the rationale for mass treatment has fuelled conspiracy theories.

A further problem with existing health education is that some of the materials used are confusing. One striking example is that posters relating to schistosomiasis show a person standing in water. A worm is shown next to this individual's heal in a circle. This is supposed to convey, through the gaze of a microscope, cercariae penetrating a foot. This is assumed rather than explained and it is all the more confusing as the foot is not magnified. As a result, the picture looks as though something like an earthworm is about to wiggle its way into the body. A similar image is used on the cover of the drug distributors' register. Not surprisingly a typical response by those who see the picture is: "we don't have worms like that!" Confusion is compounded by the fact that the primary school science textbook describes bilharzia, but only gives information about S.haematobium. In addition, the health education messages relating to soil-transmitted helminths refer to 'worms', and do not adequately distinguish between different kinds of worms. Teachers and drug distributors are expected to understand the health education message well enough to communicate the distinctions, but this was rarely found to be the case in practice. These issues were noted in 2005, and raised with district and national officials. No change was evident three years later.

It goes without saying that it would be helpful to improve the quality of health education materials, but it is also the case that health education alone will not be enough to reverse the fortunes of the programme. There are several reasons why this is the case, including the regular movement of large numbers of people across the border of Congo and, to a lesser extent, Sudan; and on-going difficulties with relying upon volunteers to distribute drugs with minimal recompense. These two issues are discussed in turn.

#### Migration patterns

Panyimur sub-county shares an international border with the Congo and the movement of Congolese and Ugandans across the border has had a profound influence on the take-up of drugs. There is no empirical data documenting the number of people moving across the border, but participant observation in Panyimur Trading Centre revealed that kin relations between Alur residing in Uganda and Congo are strong and inter-connected with substantial trading networks. Indeed, Flynn [39] has recently argued that the Alur, whether Ugandan or Congolese, perceive their identity as Alur as being much more significant than their nationality.

It is against this backdrop that hundreds of people travel from Congo to Panyimur to attend the weekly Monday market. Others come from Sudan (usually by truck) and a variety of places within Uganda, including the districts of Hoima, Masindi, Buliisa, Moyo, and Kitgum. They buy and sell fish, grain and other produce, and many of them arrive the night before and sleep in the open air, with those from Congo sleeping close to the landing sites on the shores of Lake Albert. The 10 public latrines available in the Trading Centre cannot possibly cater for the number of people arriving and there is little doubt that the regular influx of large numbers of people does little to hinder the transmission of schistosomiasis and soil-transmitted helminths.

**Figure 1:**
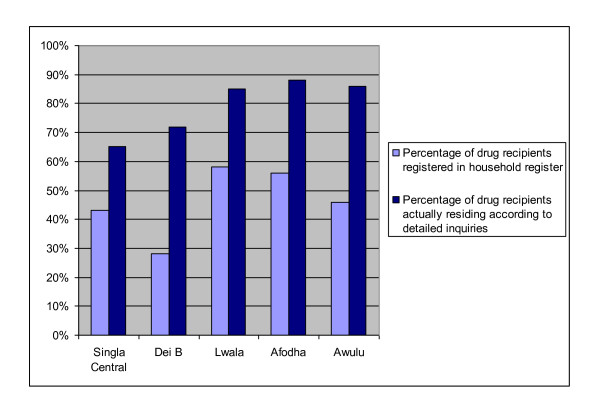
**Percentage of Drug Recipients in the Household Register Source: Adapted from Rees, **[48]**: 27)**.

Against this background, an endeavour was made to calculate the proportion of adults receiving drugs for NTDs who were residents rather than migrants. Such calculations are complex as enquiries quickly revealed that it is not always straightforward classifying someone as 'resident' or 'mobile', not least because many people who are resident in a particular village do not register with the local council. Figure 1 shows, for example, that less than a third of the actual residents in Dei B, a small village close to the border with Congo, were recorded in the household register.

**Figure 2:**
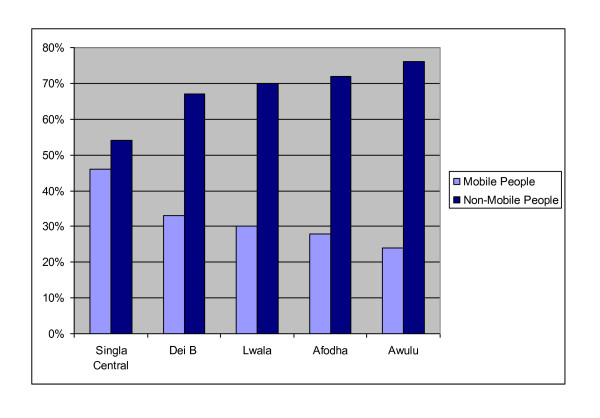
**Percentage of 'Mobile' and 'Non-Mobile' People among Drug Recipients (Source: Adapted from Rees, **[48]**: 28)**.

However, for the purposes of this research, a 'mobile person' was defined as anyone who travelled regularly, whether for one or two nights a week or one or two months a year. Figure 2 shows that a large proportion of the population were classified as mobile, with the village, Singla Central, having the highest proportion (46%) of mobile people receiving drugs. This is not surprising as the market is located in this village and large numbers of people trade here every Monday. Dei B had the second highest proportion of mobile people (33%) taking the drugs. The village is located on the Congolese border and adults frequently cross the border to buy and exchange goods. Several informants also commented on the fact that Congolese people were known to come to Dei during mass treatment programmes to take the drugs back to their country. In fact, one 18 year old trader who comes from the Congo to trade in the market every week said:

'We get [the drugs] in Dei ....We come here to buy because we can't get them in Congo ... I know someone who collects the balance of drugs after mass treatment. They hear over the radio there is going to be mass treatment, so they come'

The proportion of mobile 'adults' receiving drugs for NTDs was 30%, 28% and 24% in the villages of Lwalla, Afhoda and Awulu respectively. Almost certainly, this is due to the fact that these villages are located further away from the border with Congo.

The influx of migrants at times of drug distribution and, in accord with government policy, the willingness of drug distributors to provide the drugs to such people (many of whom are close relatives who live on the Congolese side of the border) explains why the number of drugs distributed in Panyimur is high, but does not reach all of the locally resident population. In several villages, more than half of the surveyed households did not receive drugs in 2007. The ramifications of this are significant: high rates of re-infection for S.mansoni were recorded by SCI in primary schools in the sub-county. That is, re-infection rates of 76% and 90% were recorded among school children attending Panyimur Primary Schoool and Nyakagei Primary School in 2008 respectively; and there is every reason to suppose that re-infection will continue unabated until the issue is addressed. A further difficulty concerns the reliance upon unpaid drug distributors, to which attention now turns.

#### On-going difficulties with voluntary distribution

The National Control Programme for the Integrated Treatment of NTDs relies upon volunteers to distribute drugs to adults within their 'community'. This approach assumes that communities are cohesive and that drug distributors have both the time and inclination to undertake the required tasks. Research in Panyimur highlighted the hazards of this approach. These include the following: first, the majority of drug distributors said they felt 'too poor' to work without remuneration. The following quotes capture this point:

"There is no incentive to work .. there are no uniforms, no bicycles for transport."

"We are not paid ... we cannot work well .. I have firewood to gather [from the hills], water to collect [from the lake] and six children to feed. It is too far to walk to a homestead that is 2 or 3 kilometres away at the end of the day."

"I shall give the tablets out, but refuse to register them .. that is the right option for me because the Ministry of Health have money but they refuse to release it."

District officials echoed these concerns, albeit in a different way. To quote:

"They [the drug distributors] are running out of patience ... I am worried for the next round of treatment ... ah, this sitting allowance is very little."

"The spirit of volunteerism has gone completely ... they are not realising the benefits."

A second difficulty is that the channels for distributing drugs overlooks gender relations and assumes that the benefits of free treatment over-ride other concerns. This is not the case. Participant observation fieldwork with young, female drug distributors, for example, revealed that it is impossible for a woman to insist that an older man swallows his praziquantel tablets (or any other tablets) under her gaze. In all cases, the men concerned put the tablets in their pocket, to be taken at a time of their choosing.

Third, the rationale for the provision of free treatment on a regular basis has not been communicated to drug distributors in a convincing way. As a result, they are not able to answer questions and concerns expressed by those who are meant to be swallowing the tablets. These include the following: 'why is it necessary to take praziquantel if you feel well?'; 'is praziquantel for prevention or cure?'; 'why are tablets being allocated by height rather than weight?'; 'what is the point of taking praziquantel if re-infection is almost immediate?'; 'why is it necessary for someone to take praziquantel if they do not have any sign of infection?'

#### Are there prospects of drug uptake in Panyimur improving?

In spite of on-going concerns about side-effects, inaccurate and ineffective health education, migration, and the current system of relying upon voluntary drug distributors, there were some indications that drug uptake may improve over time, without external intervention. This is because villagers are making their own empirical observations of the benefits of treatment. By way of illustration, a 59 year old male from Kidi Achoka, Ganda Parish described how he had a swollen stomach since the 1980 s but feared to take the drugs. Then, in 2005, he was drunk and decided to swallow them. He had serious vomiting, skin rashes for two weeks and he ended up going to hospital. Here, the doctor told him that he was suffering from the side-effects of the tablets rather than from a bad sickness. He subsequently became fit and strong. To quote:

"I used to suffer from blood in the stool and abdominal pain before I started taking the drugs, but after 2005, I feel I am relieved from that pain because I no longer suffer from these illnesses ... this is why I cannot deviate or hesitate from taking those drugs."

Thus findings in 2008 suggested that there were grounds for optimism, albeit small, as conspiracy theories were less prevalent, and political and economic changes occurring at a national and district level were beginning to positively affect day to day life in Panyimur. Nevertheless, few people are willing to swallow tablets if they feel they have insufficient food to mitigate the side-effects and these small changes cannot be equated with the emergence of a demand for treatment.

#### The changing political and economic context

The generally poor levels of adult drug uptake in Panyimur and slight improvement noted in 2008 also need to be understood in relation to political and economic developments in the area. In 2005, life in Panyimur was affected by insecurity. Sandwiched between the upheavals in the Democratic Republic of Congo and the on-going war between the Ugandan government and the Lords' Resistance Army [LRA] to the east, economic activity was thwarted. The situation was exacerbated by a lack of rain; dwindling fish stocks in Lake Albert; receding forests; and an apparent disinterest on the part of the government to invest in services and infrastructure. Indeed, these concerns contributed to widespread complaints about the government deliberately pursuing policies to ensure that the Alur, (the dominant ethnic group in Panyimur), remained poor and marginal. Resentment against the government was frequently expressed in terms of a north/south divide within the country and concerns about side-effects arising from treatment with praziquantel were on numerous occasions explicitly linked with accusations about government officials wanting to reduce the rate of population growth in the district. Such views were not just expressed in private, but also at focus group discussions and public gatherings.

However, considerable change occurred after 2005. Some of the most important include the following: a tarmac road running from Karuma to Pakwach was completed in 2007. This had a quick and beneficial impact on the economy at a district level, and this was visible in Panyimur (which is about 45 minutes drive away from Pakwach). Diesel, for example, is now for sale in Panyimur and there are increasing credit opportunities for local people, with district officials in Nebbi town even borrowing from local micro-finance groups in Panyimur. Some of these changes are also linked to the government making a concerted effort to gain political support in the district during the Presidential election campaign of 2005-2006. This paid off as they secured 47% of the votes - in contrast to their nearest rival, the Forum for Democratic Change, which secured 43% of the vote. Panyimur's sub-county's local council chairman (known as LC3) is now the ruling party's chosen candidate to become the next local council chairman for Nebbi district (LC5). Although various local tensions remain, notably with respect to the activities of soldiers and police on market days, general attitudes towards the government have become much more positive. One consequence of this shift in attitude was that in 2008 the provision of drugs through government structures was treated with less suspicion than was the case in 2005. Rainfall was also good that year, and food supplies not as scarce. In addition, the LRA retreated into southern Sudan and the relative stability this afforded was welcomed. These changes in Panyimur corresponded with improved responses to drug take up among some of those interviewed, although other problems noted above remained. Also, in 2009 the rains were poor and issues of food scarcity resurfaced. Expectations have been raised about the projected local benefits of oil discoveries in and around Lake Albert, but this has also led to a dramatic rise in the value of land. Concerns and complaints about government officials, especially at district level, are again expressed quite widely, and ongoing drug distribution in mid 2009 showed every sign of again achieving low uptake rates.

### Why is MDA acceptable in Moyo and Adjumani districts, and why does the uptake of drugs among adults vary so much between locations within the districts?

The uptake of drugs for the treatment of bilharzia and soil-transmitted helminths was higher at selected sites in Adjumani and Moyo districts compared to Panyimur, Nebbi district. This finding is particularly interesting as the proportion of informants reporting side-effects was not markedly less (65% and 68% compared to 70%). Differences in the mode of drug delivery could not be discerned nor was any greater effort made at communicating ideas about appropriate treatments for NTDs to local council chairmen, drug distributors and villagers. Although impossible to quantify, it is likely that differences in the take up of drugs in these nearby areas of north-western Uganda can be attributed to historical differences, while wide variations in self-reported uptake between sites within the districts can largely be explained by more recent events.

Both Moyo and Adjumani districts are predominantly populated by Madi people and historical research suggests that the Madi might be particularly amenable to the roll out of MDA, at least compared to other parts of north-western Uganda. Briefly, for most of the Protectorate period, Moyo and Adjumani districts were administered as Madi sub-district and were designated as a closed location. This meant that movement into and out of the sub-district was controlled. Even labour migration to the south of the district was restricted. There were two reasons for this: first, in the early years of the Protectorate administration, Madi sub-district was perceived by the British administration as a place affected by revolutionary Islam. It had been the location of Mahdist forts in the nineteenth century, heavily affected by slavery and ivory trading. Until the 1920 s, much of the local administration took place through chiefs in Arabic. A concerted attempt was made to reverse such trends and Italian missionaries were actively encouraged to promote Catholicism. This proved to be a remarkably successful strategy and Catholicism became a powerful force. In fact, church sermons remain profoundly influential in shaping ideas about moral probity. This relates directly to health care in that the church has persistently challenged 'pagan' beliefs, and promoted ideas about physical wellbeing that sets aside ideas about ancestral spirits in preference to biomedical therapies.

A second reason for keeping Madi sub-district a closed area was because it was seen as an epicentre for the spread of sleeping sickness. From the 1920 s, large parts of the population were moved and re-settled close to roads and administrative locations. In fact, populations inhabiting tsetse infested bush zones (such as in Lefore to the west of Moyo town and swathes of Adjumani district to the south of Adjumani town) were forcibly removed. Sleeping sickness control measures remained in force throughout the protectorate period and were re-introduced in the 1980 s following the return of thousands of Madi from Sudan (where they had fled first the fighting in Uganda and subsequently the fighting in Sudan). In collaboration with international agencies such as Medecins Sans Frontier, the Ugandan government introduced a mass testing and treatment programme in the late 1980 s, which had the effect of reversing a rising prevalence rate. To a large extent, other mass treatment programmes such as immunization campaigns have been affected by this history of successful disease control and have elicited a positive response from the population.

An effect of these policies was that by the end of the Protectorate period, the Madi were perceived by British officials as a uniquely malleable and responsive population, with a willingness to collaborate fully in public policies. They were also perceived as having a remarkable commitment to education. To a large extent, this legacy has survived the political turmoil following independence. Although Moyo and Adjumani district have been affected by these problems, it is striking that while certain Madis have been active in political life, Madi people as a whole have tended to be accepting of government authority, irrespective of the nature of the government in power. As in Panyimur, it is common to hear complaints about being "the forgotten people," but the kinds of conspiracy theories that circulate in other locations in the north are largely absent. Indeed, district officials, local vector control staff and other community leaders would often emphasise this point, stating that whereas at other locations people are always telling stories about how certain kinds of drugs are introduced for allegedly pernicious purposes (such as to secretly control the population), this is rarely the case in Moyo and Adjumani districts.

The positive response by the Madi to MDA in Moyo and Adjumani districts can be attributed to the following: first, the Catholic Church is very influential and actively supports a range of public health initiatives such as immunization programmes, child health days and endeavours to control NTDs. As a result, there is widespread enthusiasm for MDA. Second, the long history of disease control in this part of Uganda, notably the mass treatment programmes that were instigated to contain and control sleeping sickness, dating back to the Protectorate period. In contrast to Panyimur, there was no question of MDA being actively resisted. The following quote captures the willingness of Madi throughout Adjumani to receive free drugs for the treatment of bilharzia (schistosomiasis), filaria (lymphatic filariasis) and other worms (including soil-transmitted helminths):

"I have never heard of people refusing the tablets ... I was told to buy the drug after testing positive [for bilharzia] but I could not afford them so I took the ones distributed to my home ... I don't know the name of the drug," (a 25 year old male from Ogolo IDP camp.)

Even people who were aware of the side-effects expressed a willingness to receive free treatment. Thus, a 76 year old male from Melehwe IDP camp said: "People say that when it disturbs you it means you have the worms and you should continue with the tablets so that the worms can reduce in the body."

Similarly, a 45 year old female from a different IDP camp in Adjumani district who took praziquantel in 2006 and 2007 with side-effects said: "I will continue to take it until I die;" and a 20 year old male from Ogollo IDP camp who reported taking praziquantel five times said: "some say it makes you feel drunk and can make you mad." Such talk did not deter him from taking the drugs.

There were, however, considerable differences in self reported drug take-up between sites in the two districts. To a large extent that is explained by the effects of insecurity and the responses to the needs of displaced populations. In recent years, Adjumani and Moyo districts have been more directly affected by warfare than the population of Panyimur in Nebbi district. In 2008, for example, many parts of Moyo and Adjumani districts were affected by the presence of large numbers of Sudanese refugees, and the World Food Programme were providing food relief to the local population as well as Sudanese refugees. Primary schools at all the sites where research occurred in Moyo and Adjumani districts, for example, were assisted by a school feeding programme. This was still continuing in 2008 but was about to be terminated as many Sudanese were being repatriated as part of the Sudanese Comprehensive Peace Agreement. The districts have also been affected by the insecurity of surrounding areas and occasionally by incursions by a variety of military forces. The new road between the riverine locations of Laropi and Dufile running up the Metu mountains to Moyo town remains partially finished as one of the Chinese workers was killed in an attack by the LRA. In Adjumani district, which is unprotected by the River Nile, and located on one of the main LRA routes into the war zone of northern Uganda, the population has been particularly vulnerable. Indeed, one of the last LRA attacks inside Uganda occurred in the displacement camp at Ogolo in 2006. Much of the population of Eastern Adjumani district had been concentrated in this camp, located very close to the Sudan border and in sight of the Sudanese town, Nimule. For a large number of the residents, this has meant a shift from an inland location to one close to the Nile. It is for this reason that it was chosen as one of the fieldsites.

High uptake was recorded in both Ogollo and Malehwe internal displacement camps in 2007 and 2008. Life is highly regulated in these camps, with clear rules and regulations enforcing many aspects of social life. The concentration of large numbers of people into a relatively small space makes it relatively straightforward for drug distributors to distribute drugs, and MDA occurs in a context where international assistance from a range of donors is welcomed and trusted. Indeed, many would not be able to survive without such assistance. Similarly, the lower rates of drug uptake recorded in 2005 and 2006 reflect the fact that life was greatly affected by the political and economic insecurity generated by the movement of the LRA. Many of those who accepted the drugs in 2007 and 2008 were simply not offered the drugs in 2005 and 2006.

A further reason for variations in self-reported drug take up relate to widespread knowledge of side-effects. The fact that a need to eat before swallowing praziquantel is well known has meant that the uptake of drugs has been greatly influenced by the activities of the World Food Programme. In 2007, food was widely distributed to Sudanese refugees as well as Ugandan Madi accommodating them in their villages (notably Adjujo and Arraa central.) The repatriation of Sudanese refugees in March 2009 led to the withdrawal of the World Food Programme to Sudan. As a result, the uptake of drugs among school children and adults dropped at several locations as teachers were not willing to distribute drugs in the absence of food and adults were similarly not willing to swallow them.

In Arra Central, for example, drug uptake fell to 5% in 2008 as large numbers of Madi refused to accept treatment without food. It should also be added that there were serious shortages of food in Adjumani and Moyo between July and September 2009 as international agencies were buying up food in these two districts to distribute in Sudan. Consequently, food that would otherwise have been for sale in Ugandan markets was no longer present. The spiralling cost of food in northern Uganda is generating considerable hunger - though it was beyond the remit of this research to verify this observation empirically.

It is important to note that self-reported uptake of drugs exceeds those documented in the NTD Ministry of Health registers and, almost certainly, is much more accurate. There are two reasons for this: first, the system for monitoring the distribution of drugs has effectively broken down with the introduction of the 'new' NTD registers in 2007/8. That is, community drug distributors, feel completely confused by the fact that there are now two registers in circulation: one for monitoring the distribution of ivermectin and albendazole for the treatment of lymphatic filariasis and onchocerciasis and one for monitoring the distribution of all NTD drugs including ivermectin and albendazole. Unsure as to whether they should copy the names of people receiving ivermectin and albendazole from the lymphatic filariasis/onchocerciasis register into the 'new' NTD register, when they are only receiving one round of ivermectin and albendazole a year, they end up writing down the names of only a few of those who actually receive treatment and there is an element of serendipity as to whether the names recorded go in the 'old' lymphatic filariasis/onchocerciasis register or the 'new' NTD register. Second, population figures reported to the district have been persistently exaggerated, often by as much as three times the actual population. This persistent exaggeration has the explicit aim of securing additional food aid, but it also means that it is unhelpful to gauge the proportion of the people receiving MDA by comparing the number of people recorded as taking drugs for NTDs with census data held at the district level.

### Why has control of NTDs been apparently successful in Busia district?

The political, economic and social context of treatment in Busia district differs markedly from that outlined for north-western Uganda. It is located at the opposite end of the country from the other research sites, in a region that has benefitted substantially from the relatively high economic growth of Uganda since the late 1980 s. Although there have been border tensions with Kenya and also violent incidents at times of elections, the area has not been directly affected by armed insurgencies or refugee influxes. The district has attracted substantial private and governmental investment, and its location at the most busy border crossing with Kenya, Uganda's most economically successful neighbour, as well as its ready accessibility from Kampala, has given it a different national visibility and political significance to areas of the northwest. Indeed, it is frequently held up by government officials as a model district, with a committed local administration and a place where public health programmes are run effectively.

Significantly, in 2009, a large new fish processing plant in Lumino sub-county was operating close to the lake shore, and a high grade landing site and market was being constructed nearby. A tarmac road was about to be constructed, linking these projects with Busia town. A new bridge and road was also planned, giving more direct access to the main highway to Kenya. These initiatives were being implemented at the same time as new public health policies aimed at cleaning up the district, and regulating access to the lake were being enforced. The implications of these initiatives are discussed further on.

Of all the districts studied in Uganda, Busia was found to be the most successful at NTD control. This can be partially explained by the relatively high take up and consumption of tablets. However, it is not the whole story. In common with the Madi and Alur of north-western Uganda, it was found that few of those interviewed had much understanding of how infections with bilharzia and other kinds of parasitic worms occurred. Here, as in the northwest of Uganda, infection with 'bilharzia' is generally connected with water sources in a vague way, and almost everyone mentioned the drinking of dirty water. So a reported decline in infection from S.mansoni by the Ugandan Ministry of Health does not correlate with better local knowledge. Instead, as with the north-western part of the country, political and economic changes shed light on the situation.

Busia is widely perceived as a 'model' district, and the government has a visible and active presence within the district. The security of the new fish processing plant in Lumino sub-county is being managed by a private security firm; and this firm, closely associated with the Beach Management Unit, is helping to regulate access to the lake. In 2007, for example, more than 3/4 of registered fisherman had their licenses revoked and fishing in the vicinity of the landing sites is now tightly controlled by the Beach Management Unit. This has significantly reduced the number of people accessing the lake on a regular basis, but it has also had ramifications for the population as a whole as large numbers of adults used to be involved, if not in fishing, then fish processing and/or the provision of services to fishermen at landing sites. Although it was impossible to acquire detailed empirical data in the time available, long-standing residents noted that the total number of people living close to the landing sites had dropped dramatically in recent years.

Running alongside a commitment to clean up beach landing sites and the lake shore more generally, public health acts have been used to enforce hygiene behaviours. These include the enforcement of a 5,000 shilling fine for any household that does not have a pit latrine, as well as a 5,000 shilling fine for anyone caught urinating or defecating in the lake and a 3,000 shilling fine for anyone caught bathing in the water.

A further difference to the scenarios described in Panyimur, Nebbi district and Moyo and Adjumani districts, is that food is generally available. This means that those taking praziquantel are generally doing so after eating, reducing the risks of serious side effects. The occurrence of relatively minor side-effects, alongside the visible presence of the state attempting to improve roads, develop 'ecological' fishing etc has undoubtedly contributed to the prevailing view that the mass distribution of drugs by district health staff is beneficial to local people.

While it is impossible to assess the extent to which the decline in S.mansoni infection is the result of MDA rather than the imposition of public health acts restricting access to the lake, it is also important to note that there have been serious consequences for the neighbouring district, Bugiri. Research carried out in 2009 suggests that Busia has to some extent passed the difficulties of controlling NTDs to other areas. Findings on self-reported uptake of praziquantel and albendazole among adults from Banda and Sigulu sub-counties of Bugiri indicate that 34% received these drugs in 2008 and 43% received these drugs in 2007. These figures are based on a 10% sample of randomly selected households from 5 villages in Banda sub-county and 5 villages in Sigulu sub-county. The selected villages in Sigulu sub-county were located on Sigulu and Lolwe islands, the former being 40 minutes from the mainland and the latter being 2 hours and 40 minutes by boat from the mainland. There has been no MDA in 2009 and limited MDA in 2008 as the district vector control officer has been seriously ill for 18 months and no effective replacement has been found. As a result, only 13 of the 39 villages received MDA in 2008.

Ugandan Ministry of Health data documenting the prevalence of S.mansoni in Bugiri district suggested that in 2007 the prevalence was 46%. It is not at all clear what type of impact MDA may have had on the prevalence of infection, but it is unlikely to have been as effective as in Busia district. This is not only due to the relatively low coverage of MDA, but also to the fact that 18 of the 39 villages in Bugiri district are located on the lake shore and water and sanitation facilities are very limited (in part because the soil is often too light to support pit latrines and the water from bore holes is too salty to drink). In addition, no attempt has been made to enforce by-laws that interrupt transmission. In Sigulu sub-county, there are 142 inhabited islands with logistical and budgetary reasons often being cited as explanations for the dearth of MDA. These need to be overcome as 98% adults in the sub-county reported that they had not received any treatment in the last two years and some of those that had received treatment had had to pay local councils for the drugs. Investigation of the clinic on the biggest island Sigulu, revealed that there were 5 unopened tins of praziquantel, suggesting that the picture is much more complex than overcoming logistics and budgetary constraints. Census data is hard to come by, but there is clearly considerable migration into the area. At one landing site on Lolwe Island, for example, it was reported that the number of registered fisherfolk had increased from 328 to around 1000 following the restrictions imposed in Busia district.

In sum, there is nothing particularly remarkable about drug delivery strategies in Lumino sub-county, Busia district and self-reported uptake is not as high as in parts of Adjumani and Moyo district. Drug distributors, whilst willing to take the time to distribute drugs, are not exceptionally well motivated. Registers are poorly kept and there is no endeavour to ensure that drugs are always swallowed. However, the low recorded prevalence of S.mansoni demonstrates that a political agenda to develop 'ecological' fishing, in combination with a willingness to enforce public health acts, to ensure that Busia sustains its reputation as a flagship district has played a major role in contributing to the decline in prevalence.

## Conclusions

The comparative study of drug uptake in three districts in north-western Uganda and two districts in south-eastern Uganda has highlighted the differential uptake of drugs between districts as well as variations within districts over time. Insights emerging from ethnographic research are used to interpret quantitative data on drug uptake as well as parasitological data detailing the prevalence and intensity of infection. In so doing, it has become clear that a single policy formulated by the World Health Organisation, in consultation with advisors from London, Seattle, Washington and staff from the Ministry of Health in Uganda, cannot be rolled out in a uniform way across the country; and it will inevitably be influenced by the political, economic, historic and social context in which it occurs. This, in turn, affects responses to MDA as well as rates of re-infection. The implications of this finding are wide-ranging and they are discussed below.

Before doing so, it is important to draw attention to the point that the data presented in this paper suggest that a large majority of adults at field sites in north-western and parts of south-eastern Uganda have had the opportunity to receive drugs, free of charge, for schistosomiasis and soil-transmitted helminths since 2004. In some districts, such as Adjumani, reported take-up in particular years was very high, and most adults will have had the opportunity to take treatment on that occasion. Elsewhere, such as parts of Nebbi District, no single distribution in any given year reached a similarly high percentage, but almost every long-term resident interviewed had been offered treatment at least once. Although not a primary focus of our research in Uganda, this was also the case where treatment was investigated for lymphatic filariasis and onchocerciasis. While coverage has not reached the targets initially planned for high take up of repeated treatments, this is still a considerable achievement.

To move forward from this point necessitates lessons being learned from what has worked and what has failed. It requires the kind of information reported in this paper. Points arising from the findings include the following. First, there is a need to put in place an adequate system for monitoring as the programme cannot run effectively without identifying difficulties quickly and easily. Relying upon volunteers to keep a detailed record of drug uptake year on year, whilst simultaneously increasing their work load with minimal remuneration, is generating large quantities of inaccurate data. Opportunities are being missed by not collecting and analysing robust information about drug uptake and relating this to systematic assessments of disease prevalence and intensity of infection.

Second, these problems are exacerbated by the fact that USAID/RTI currently fund and take primary responsibility for implementing the integrated NTD programme. It is run as a parallel structure located within the Ministry of Health, with rounds of funding linked to 'successful' implementation. 'Off the record', many of those involved with MDA are well aware that there are difficulties with the programme, but fear that the publication of more objective evaluations will lead to the withdrawal of funding and free drugs, rather than a better programme. They find themselves in an impossible position.

Third, at all research sites, health education is poor and where it does occur, it assumes that people only need selective information. As a result, local understandings of the transmission of parasitic infections are confused and information about appropriate health-seeking behaviours is muddled. For example, it is very common for people to attribute bilharzia to the fact that they might not have washed their hands before eating or that they did not boil the water before drinking it. In Panyimur sub-county, the limited and inaccurate health education materials fuelled conspiracy theories. Health education needs to address local concerns and anxieties. It is not just a question of producing new and more detailed booklets, though this is needed too as a matter of urgency.

Fourth, it would be helpful for district officials to enforce existing public health acts. If successes reported in Busia district provide an example to follow, it is surely that transmission can be reduced by using existing legislation to regulate behaviour - including the imposition of fines for not having a pit latrine and defecating by or in the infected water, and possibly limiting access to certain rivers and lakes.

Fifth, it would be fair to say that MDA has not yet generated a demand for treatment. While there are a few places where adults are articulating a desire for treatment (notably in villages along the River Nile in Moyo district); and while there were a few individuals at some of the other research sites articulating a demand treatment; the majority of adults in targeted areas are not pressurising district authorities and medical personnel for a continuous supply of tablets. In addition, there is no evidence to suggest that they will be willing to buy the medicines themselves once MDA comes to an end. It is hard to see how a demand for treatment can emerge as there is so much confusion about the rationale for MDA at all the research sites. Conspiracy theories in Uganda have not snowballed in the way that they have in Tanzania, where violent riots characterised local response to MDA in some locations in 2008.^5 ^Nevertheless, they exist at all sites, and in some of the distributions studied have impinged upon drug take-up. Local concerns about the rationale for MDA clearly need to be addressed more adequately than they are at present.

The findings presented in this paper resonate with those reported by anthropologists working on other vertical interventions linked to the Millenium Development Goals health objectives, such as immunization coverage in West Africa [40-42] and the control of tuberculosis in Nepal [43]. More specifically, it remains to be asked how much can be generalised from the findings presented here on schistosomiasis and soil-transmitted helminths in Uganda; and what type of insights emerge by comparing local responses to the treatment of these diseases with treatment for other NTDs.

As has been highlighted above, there is a paucity of evidence with which to answer these questions. Recently, Kabatereine has reported that MDA has contributed to the eradication of onchocerciasis at some locations in Uganda, that S.haematobium may also be eradicated soon, and lymphatic filariasis is on track for elimination by 2020 (Kabatereine, personal communication in October, 2010). These are encouraging observations, but it would be valuable to investigate what has been achieved in detail at the local level, not least because Katabarawa and colleagues drew attention to problems arising in onchocerciasis control at their Ugandan research sites in the 1990 s [16,17]. Also, research undertaken by Parker, Allen and Hastings on the roll out of MDA in Tanzania for S.haematobium, S.mansoni, lymphatic filariasis, soil transmitted helminths and onchocerciasis suggests that comparable issues have arisen to those observed in Uganda with respect to S.mansoni. These include a tendency to persistently overstate drug uptake which at some locations was found to be very low indeed (see Hastings [44] and M Parker and T Allen 'social responses to mass drug administration in northern coastal Tanzania', report submitted to the SCI, Imperial College, London, April 2008). Research on a number of different NTDs in Tanzania additionally raises a point that has not been discussed explicitly with respect to Uganda, but would have emerged more clearly if MDA for a similar range of infections had been studied - namely, that NTDs are not experienced as a homogenous category of infections and it is misleading to treat them as if they are.

Information presented here has related primarily to S.mansoni and soil-transmitted helminths, but most of the problems highlighted relate to the control of the former rather than the latter. To a large extent this is connected with the subjective perceptions of those being treated. Everyone interviewed recognised that a human being - or a domestic animal - could be infected with worms. The worms can sometimes be seen in the stool. It is also known that certain kinds of local and manufactured medicines result in a purging of worms from the body. This, too, is directly connected with experience of having watched it happen. By contrast, S.mansoni, requires a person who is likely to think he or she is healthy accepting the idea that it is possible to be infected without feeling ill. This, in itself, contradicts health care messages about taking drugs without a proper diagnosis. It also requires a large degree of trust in those delivering treatment and confidence that they know what they are doing. In many situations, such trust and confidence is lacking.

Research carried out in Tanzania has found a similar difficulty with respect to lymphatic filariasis. Here, too, a leap of faith is required by those on the receiving end of MDA as it is important to be treated before symptoms become manifest. Infection with S.haematobium, on the other hand, is often characterised by the visible symptom of reddy-brown urine among children and young adults. As a result, there was widespread recognition in some locations that treatment might be necessary. However, this was not found to be the case at all study sites. In these places, the fact that the symptom of bloody urine is not present in most infected adults was linked to a perception that it is a harmless condition - one that only affects children, and which they grow out of it even if they remain untreated. This, in turn, fuelled scepticism and conspiracy theories about why free treatment was being offered. Lack of communication by those distributing tablets - and specifically inadequate explanations for why school children were being made to take tablets at school by their teachers - was a primary cause of the above mentioned riots in 2008.

None of this means that the MDA approach cannot work as, in some places, it already has. But, as things stand, international assistance and funding has too many strings attached. It is being spent too quickly and it is as likely to weaken health systems as it is to strengthen them. It is surely unrealistic to assume that a vertical programme, delivered through mechanisms that are effectively separate from those developed within government ministries and poorly informed about local circumstances, will improve the long-term health of populations that, for the most part, are politically and economically marginal. There are reasons why these people have tended to be overlooked and why they may distrust the motivations of those instructing them to take tablets. Those reasons cannot just be wished away. There is a requirement for a serious rethink, including more sensible and more closely evaluated targets, and, above all, responses that are sensitively tuned to particular circumstances. To persist with the current "one size fits all" approach is to fail those most in need of assistance.

## Competing interests

The authors declare that they have no competing interests.

## Authors' contributions

MP and TA contributed in equal part to all aspects of this paper. Both authors read and approved the manuscript

## Appendix

^1^Considerable emphasis was placed upon the need to create a demand for treatment in the early years of the programme. It is re-iterated in the recent process evaluation of Schistosomiasis Control published by staff from SCI, Imperial College and the Ugandan Ministry of Health. To quote: 'It was intended that the initial support from SCI would reduce the disease burden associated with helminth infection and provide districts with experience in controlling disease, thereby creating a demand for treatment and sustainable government financial and political support' [[32]:1].

^2 ^This focus was due to our link with SCI, Imperial College and the Vector Control Division, Ministry of Health, Uganda. In addition, schistosomiasis has been a long-standing research interest of one of the authors (see, for example, Parker [45]; Parker [46] and Parker [47].)

^3 ^This paper is based upon fieldwork funded by the Bill and Melinda Gates Foundation as part of a grant to SCI, Imperial College. London. Ethical clearance was passed by the ethics committee of Imperial College for surveillance and monitoring activities for the evaluation of national control programmes for the control of NTDs. We worked closely with staff from the Ministry of Health, Uganda and obtained additional research clearance at a national and district level.

^4 ^The five selected villages were chosen to capture the variety of villages in the sub-county. Singla Central is the most densely populated trading area within Panyimur as the market is located here and attracts visitors from Sudan, the Democratic Republic of Congo, and Kenya. Dei B is located at the border between Uganda and the Democratic Republic of Congo. Here, it is interesting to note that children living close to the border in Congo cross the border every day to go to primary school in Nyakagei Parish, Panyimur. Afhoda lies to the west of Singla Central whereas Awulu is located near the shores of Lake Albert between Singla Central and Dei B and Lwalla is located in the hills rising up from the lake.

^5 ^These events were closely observed by one of our PhD students, Julie Hastings, who had to be rescued from her field research site by Tanzanian police [44]. The events were reported in the Tanzanian media, and have led to retrenchment in some of the Tanzanian MDA operations.

## References

[B1] Brady MA Hooper PJ Ottesen EA Projected benefits from integrating NTD programs in sub-Saharan Africa TRENDS in Parasitology 2006 22 7 285 291 10.1016/j.pt.2006.05.007 16730230

[B2] Engels D Savioli L Reconsidering the underestimated burden caused by neglected tropical diseases TRENDS in Parasitology 2006 22 8 363 366 10.1016/j.pt.2006.06.004 16798088

[B3] Fenwick A Molyneux D Nantulya V Achieving the Millennium Development Goals Lancet 2005 365 1029 1030 15781095 10.1016/S0140-6736(05)71134-X

[B4] Fenwick A New initiatives against Africa's worms Transactions of the Royal Society of Tropical Medicine and Hygiene 2006 100 200 207 10.1016/j.trstmh.2005.03.014 16343572

[B5] Molyneux DH 'Neglected diseases' but unrecognized successes - challenges and opportunities for infectious disease control Lancet 2004 364 380 383 10.1016/S0140-6736(04)16728-7 15276399

[B6] Molyneux DH Hotez PJ Fenwick A Rapid-impact interventions: how a policy of integrated control for Africa's neglected tropical diseases could benefit the poor PLOS Med 2005 2 11 e.336 10.1371/journal.pmed.0020336 PMC1253619 16212468

[B7] Manderson L Aagaard-Hansen J Allotey P Gyapong M Sommerfeld J Social research on neglected diseases of poverty: continuing and emerging themes PLoS Neglected Tropical Diseases 2009 3 2 e332 10.1371/journal.pntd.0000332 19238216 PMC2643480

[B8] Hotez PJ Forgotten people, forgotten diseases: the neglected tropical diseases and their impact on global health and development 2008 Washington: ASM Press

[B9] Hotez PJ Fenwick A Savioli L Molyneux DH Rescuing the bottom billion through the control of neglected tropical diseases The Lancet 2009 373 1570 75 10.1016/S0140-6736(09)60233-6 19410718

[B10] Kabatereine NB Fleming F Nyandini U Mwanza JCL Blair L The control of schistosomiasis and soil-transmitted helminths in East Africa TRENDS in Parasitology 2006 22 7 332 339 10.1016/j.pt.2006.05.001 16713357

[B11] Kabatereine NB Brooker S Koukounari A Kazibwe F Tukahebwa EM Fleming FM Zhang Y Webster JP Stothard JR Fenwick A Impact of a National Helminth Control Programme on infection and morbidity in Ugandan schoolchildren Bulletin of the World Health Organization 2007 85 91 99 10.2471/BLT.06.030353 17308729 PMC2174620

[B12] Knopp S Mohammed KA Rollinson D Stothard RJ Khamis SI Utzinger J Marti H Changing patterns of soil-transmitted helminthiases in Zanzibar in the context of national helminth control programs The American journal of tropical medicine and hygiene 2009 81 6 1071 8 10.4269/ajtmh.2009.09-0377 19996439

[B13] Standley CJ Adriko M Alinaitwe M Kazibwe F Kabatereine NB Stothard RJ Intestinal schistosomiasis and soil-transmitted helminthiasis in Ugandan schoolchildren: a rapid mapping assessment Geospatial health 2009 4 1 39 53 19908189 10.4081/gh.2009.209

[B14] Yu D Cost-effectiveness analysis of the impacts on infection and morbidity attributable to three chemotherapy schemes against Schistosoma Japonicum in hypoendemic areas of the Dongting Lake region, China Southeast Asian Journal of Tropical Medicine and Public Health 2002 33 441 457 12693575

[B15] World Health Organization *Working to overcome the global impact of neglected tropical diseases*, First WHO report on neglected tropical diseases Geneva 2010

[B16] Katabarawa MN Mutabazi D Richards FO Controlling onchocerciasis by community-directed, ivermectin-treatment programmes in Uganda: why do some communities succeed and others fail? Annals of Tropical Medicine and Parasitology 2000 94 343 352 10.1080/00034980050034590 10945044

[B17] Katabarwa MN Richards F Community-directed health (CDH) workers enhance the performance and sustainability of CDH programmes: experience from ivermectin distribution in Uganda Annals of Tropical Medicine and Parasitology 2001 95 275 286 10.1080/00034980120072260 11339887

[B18] Amazigo U The challenges of community-directed treatment with ivermectin (CDTI) within the African Programme for Onchocerciasis Control (APOC) Annals of Tropical Medicine and Parasitology 2002 96 S41 S58 10.1179/000349802125000646 12081250

[B19] Parker M Allen T Hastings J Resisting control of neglected tropical diseases: dilemmas in the mass treatment of schistosomiasis and soil-transmitted helminths in north-west Uganda Journal Biosocial Science 2008 40 161 181 10.1017/S0021932007002301 17761005

[B20] Gryseels B Polman K Clerinx J Kestens L Human schistosomiasis Lancet 2006 368 1106 1118 10.1016/S0140-6736(06)69440-3 16997665

[B21] Coulibaly Y Cavalli A van Dormael M Polman K Kegels G Programme activities: a major burden for district health systems? Tropical Medicine and International Health 2008 13 12 1430 1432 10.1111/j.1365-3156.2008.02174.x 18983273

[B22] Utzinger J Bergquist R Shu-Hua X Singer BH Tanner M Sustainable schistosomiasis control - the way forward Lancet 2003 362 1932 1934 10.1016/S0140-6736(03)14968-9 14667754

[B23] Kolaczinski JH Kabatereine NB Onapa AW Ndyomugyenyi R Kakembo AS Brooker S Neglected tropical diseases in Uganda: the prospect and challenge of integrated control TRENDS in Parasitology 2007 23 485 493 10.1016/j.pt.2007.08.007 17826335 PMC2682772

[B24] Dansio-Appiah A de Vlas SJ Interpreting low praziquantel cure rates of Schistosoma mansoni infections in Senegal TRENDS in Parasitology 2002 18 125 129 10.1016/S1471-4922(01)02209-7 11854090

[B25] Lammie PJ Fenwick A Utzinger J A blueprint for success: integration of neglected tropical disease control programmes Trends in Parasitology 2006 22 7 313 21 10.1016/j.pt.2006.05.009 16713738

[B26] Gryseels B Mass treatment for worms is mistaken Financial Times 2006

[B27] Tchuente TL-A Kabatereine N Karanja D Dennis E Mass treatment for worms is the correct way forward Financial Times 2006

[B28] Yamey G The unsung hero of neglected tropical diseases: interview with Narcis Kabatereine PLoS Neglected Tropical Diseases 2009 3 12 e546 10.1371/journal.pntd.0000546 20027212 PMC2791160

[B29] Kabatereine NB Malecela M Lado M Zaramba S Amiel O Kolaczinski JH How to (or Not to) Integrate Vertical Programmes for the Control of Major Neglected Tropical Diseases in Sub-Saharan Africa PLoS Neglected Tropical Diseases 2009 4 6 e755 10.1371/journal.pntd.0000755 PMC2894133 20614017

[B30] Brooker S Kabatereine NB Myatt M Stothard JR Fenwick A Rapid assessment of Schistosoma mansoni: the validity, applicability and cost-effectiveness of the LOT Quality Assurance Sampling method in Uganda' Tropical Medicine and International Health 2005 10 7 647 58 10.1111/j.1365-3156.2005.01446.x 15960703 PMC1975759

[B31] Lubanga RGN District Stakeholders' Perceptions of bilharzia and control activities in Uganda Report for the Bilharzia Control Program, Vector Control Division, Ministry of Health, Uganda 2007

[B32] Fleming FM Fenwick A Tukahebwa EM Lubanga RGN Namwangye H Zaramba S Kabatereine NB Process evaluation of schistosomiasis control in Uganda, 2003-2006: perceptions, attitudes and constraints of a National Control Programme Parasitology 2009 136 13 1759 69 10.1017/S0031182009990709 19695107

[B33] Allen T Coming home: the international agencies and returnees in West Nile Journal of Refugee Studies 1988 1 2 166 175 10.1093/jrs/1.2.166

[B34] Allen T Bernt-Hansen H, Twaddle M The quest for therapy in Moyo District Changing Uganda 1991 London: James Currey 149 161

[B35] Allen T Bernstein H, Crow B, Johnson H Upheaval, affliction and health: a Ugandan case study Rural Livelihoods: Crises and Responses 1992 Oxford: Oxford University Press 217 248

[B36] Allen T In Search of Cool Ground: War, Flight and Homecoming in Northeast Africa *(editor)* 1996 Africa World Press/UNRISD/James Currey

[B37] Allen T The violence of healing Sociologus 1997 47 2 101 128

[B38] Allen T Trial Justice: The Lord's Resistance Army and the International Criminal Court 2006 London: Zed Press

[B39] Flynn R External constraints and the moral economy of cross-border trade Journal of East African Studies in press

[B40] Leach M Fairhead J Vaccine anxieties: global science, child health and society 2007 London: Earthscan Publications

[B41] Yahya M Polio vaccines - "No thank you!" Barriers to Polio Eradication in northern Nigeria African Affairs 2007 106/423 185 204

[B42] Renne EP The politics of polio in northern Nigeria 2010 Indiana University Press

[B43] Harper I Anthropology, DOTS and understanding tuberculosis control in Nepal Journal of Biosocial Science 2006 38 1 57 67 10.1017/S0021932005000982 16266447

[B44] Hastings J Rumours and Riots: Violent encounters in the treatment of neglected tropical diseases in the Southern Highlands of Tanzania Paper presented at an 'Anthropology in London' workshop in June 2009

[B45] Parker M Re-assessing disability: the impact of schistosomal infection on daily activities among women in Gezira Province, Sudan Social Science and Medicine 1992 35 7 877 890 10.1016/0277-9536(92)90102-V 1411688

[B46] Parker M Bilharzia and the boys: questioning common assumptions Social Science and Medicine 1993 37 4 481 492 10.1016/0277-9536(93)90283-A 8211260

[B47] Parker M Boyce AJ, Reynolds V Living with schistosomes: adaptation, accommodation or severe ill-health? Human populations: diversity and adaptability 1995 Oxford: Oxford University Press 155 173

[B48] Rees N From migration to mobility: understanding how multi-spatial livelihoods affect health interventions for the mass treatment of neglected tropical diseases in north-west Uganda MSc Dissertation. London School of Economics (in partial fulfilment for the requirements of an MSc in Development Management) 2008

